# Doctor in Medicine, and Other Papers

**Published:** 1872-08

**Authors:** 


*Doctor in Medicine: And other papers on professional subjects.* By STEPHEN SMITH, M. D. New York: WM. WOOD & Co., 1872.

We have read with much interest the papers on various subjects of which this work is composed, and with many have been highly pleased.

The book comprises fifty-eight papers on subjects of professional interest. The opening article, Doctor in Medicine, in an interesting manner presents some suggestive ideas in regard to the degree of M. D., which is so often easily gained and unworthily worn. The paper on the duty of coroners shows that that ancient institution has fallen into as much disrepute in other parts of the state as it has in Erie County.

The question of nostrum advertising, which is becoming a growing evil in our religous and secular papers, is treated in plain language, the concluding sentence of which is worthy of attention by the publishers of what profess to be papers suitable for family reading and instruction:

"*Now, if quack advertisements must go to the homes of Christian families, we say, let them be taken there as quack advertisements, and not by a messenger who professes to stand upon 'great primal Christian truths' in their distribution. We cannot think that 'the time has come for a living Christianity' thus 'to assert itself.'*"

The book is full of entertaining and suggestive reading, and comes from the pen of a physician whose position in the profession entitle his words to respect and attention.

